# Disrupted mothering in Iranian mothers with breast cancer: a hybrid concept analysis

**DOI:** 10.1186/s12905-021-01346-w

**Published:** 2021-06-05

**Authors:** Effat Mazaheri, Akram Ghahramanian, Leila Valizadeh, Vahid Zamanzadeh, Tonia C. Onyeka

**Affiliations:** 1 Students’ Research Committee, Tabriz University of Medical Sciences, Tabriz, Iran; 2 Department of Medical Surgical Nursing, School of Nursing and Midwifery, Hematology and Oncology Research Center, Tabriz University of Medical Sciences, Shariati Jonubi Avenue, PO Box 5138947977, Tabriz, Iran; 3 Department of Pediatric Nursing, School of Nursing and Midwifery, Tabriz University of Medical Sciences, Tabriz, Iran; 4 Department of Medical Surgical Nursing, School of Nursing and Midwifery, Tabriz University of Medical Sciences, Tabriz, Iran; 5 Pain Physician, Palliative Medicine Physician, Department of Anaesthesia/Pain and Palliative Care Unit, Multidisciplinary Oncology Centre, College of Medicine, University of Nigeria, Ituku-Ozalla Campus, Enugu, Enugu State Nigeria

**Keywords:** Breast cancer, Cancer nursing, Disrupted mothering, Hybrid concept analysis, Iran, Maternal role

## Abstract

**Background:**

Defining the disrupted mothering would contribute to developing strategies to support mothers with breast cancer. The aim of this study was to analyze the concept of mothering disruption using a hybrid model.

**Methods:**

The Hybrid method for concept analysis was implemented consisting of three phases: theoretical, fieldwork, and final analysis. In the theoretical phase, the literature was searched using electronic databases including PubMed, ScienceDirect, Scopus, ProQuest, Google Scholar, CINAHL, Wiley, Ovid, Magiran, and SID from 2000 to 2020. Any quantitative or qualitative studies published in English or Persian, which were focused on mothering disruption in mothers with breast cancer were included in the study. In the phase of fieldwork, 20 mothers were interviewed to explore the aspects of mothering disruption. The interviews were transcribed and analyzed with conventional content analysis. In the final phase, an overall analysis of the two previous phases was performed.

**Results:**

In the theoretical phase, the following attributes were determined: “disturbance in maternal identity and roles”, “maternal insensitivity and unresponsiveness: disconnection physically and psychologically”, “the career disruption process” and “biographical disruption”. The fieldwork phase explored three themes including “the unbalance between multiple roles”, “role failure”, and “reduced maternal sensitivity”. The final synthesis yielded that the main integrated elements of mothering disruption are “disease as threating maternal role and identity”, “inability to interpret and respond to child behaviors and needs”, and “support for transitioning from being patient toward maternal competency”.

**Conclusion:**

With a deeper understanding of the term ‘disrupted mothering’ or ‘mothering disruption’, healthcare providers will have a foundation to improve cancer care, deliver effective communication and help such mothers cross this disruption and achieve restoration of their mothering role. Future research is needed to validate this concept and explore connections with health outcomes.

**Supplementary Information:**

The online version contains supplementary material available at 10.1186/s12905-021-01346-w.

## Background

**‘**Mothering’ is as a woman’s emotional and/or physical care for a dependent child [1]. Identity of woman as a mother may be threatened morally and existentially when her ability to care for her children is disrupted due to chronic illness [2]. Mothers with cancer are distressed by the periods, when their illness, through hospitalization, fatigue or disability, prevents them for caring for their children as they normally would [3].

Disrupted mothering based on disease and its nature is experienced in different ways, from decreasing physical energy for caring for children, to physical separation and increasing disability, future hospitalization or impending death. Mothering could be disrupted even when women are physically present with their children [4].

Women with breast cancer (BC) have expressed feelings such as guilt [3], being different from other mothers [4], shame about their disease and mothering capability, lack of acknowledgement from healthcare professionals regarding their role as mother and a desire to stay alive/healthy so that they can retain their position as primary caregiver [1]. Mousavi et al. emphasize that in Iran, mothers with breast cancer are weaker than healthy mothers in terms of family functioning such as problem-solving, communication, and roles [5]. They have great difficulty in fulfilling their responsibilities regarding child welfare and the stability of family life [6]. Moreover, because of Iran’s socioeconomic status and the important role of women as mothers and primary caregivers to children BC significantly disrupts the mother’s ability to function [7].

The concept of disrupted mothering was identified by Jackson who suggested that women experience guilt and distress when their mothering role is disrupted, and that this disruption may be had serious and long-term outcomes [8]. Disruption are found to be associated more with symptoms from illness and treatment, as some women do not experience illness as disruptive if they are asymptomatic. Disruption has also been found to be contextual, with women who have a history of disruptive life events being less perturbed by an illness diagnosis [9].

It is pertinent to note that in reviewing existing literature on the subject matter, disruption seems to be an incidental concept mentioned mainly in the titles or bodies of research articles, and not framed as a distinct concept in the literature. Focus upon mothering in the context of specific diseases is valuable, especially when trying to discern the overall effect of illness on mothering. It would be therefore valuable to examine the disruptive nature of illness in the context of mothering generally [9]. However, the concept of disrupted mothering remains unexplored or elaborated upon in the literature, and has not been previously defined [1]. No study has been done on the concept of the mothering disruption in Iran. There is therefore a need for a study that deeply explores the disrupted mothering concept and this current study intends to fill that gap using a concept analysis. A concept analysis is considered an important stage of scientific development in any discipline. In fact, concepts are the constructive blocks of theory that give rise to the development of the body of nursing knowledge [10]. Concept analysis is the term used most commonly in nursing and is generally applied to the process of inquiry that examines concepts for their level of development as revealed by their internal structure, use, representativeness, and relationship to other concepts [11]. A concept analysis makes the concept practical by providing clear and transparent definition and can serve as a basis for planning, performance and evaluation of nursing training [12]. Clarifying, recognizing, and defining concepts that describe phenomena is the purpose of concept analysis [11]. Because of the highly diverse society of Iran [13], an examination of this concept in the sociocultural context of Iranian cancer care is necessary to promote positive outcomes of mothers with BC, contribute to healthcare providers clinical acumen when providing health services and fill gaps in professional knowledge as well as transfer this knowledge to nursing practice [9]. Defining the disrupted mothering as a phenomenon would contribute to developing strategies to support such mothers coping with cancer alongside providing motherly care for their children. Therefore, this present concept analysis was carried out with the aim of achieving an in-depth understanding of the concept of disrupted mothering in the social and cultural context of Iranian cancer care for mothers with BC.

## Method

### Design

Several methods for development and analysis of concepts are available. One of the most widely used methods in nursing is the McEwen and Wills’ Hybrid Model that refines concepts [11, 14], eliminates ambiguity in concepts and investigates them in current context and knowledge domain [15]. This method consisted of three phases in this study: theoretical, fieldwork and final integration phase [11]. The initial phase called the theoretical phase examined the literature for the concept of mothering disruption. Guidelines for Systematic Reviews and Meta-Analyses (PRISMA) was utilized (Additional file 1). At the fieldwork stage, qualitative methodology was employed to obtain data which was analyzed and consolidated criteria for reporting qualitative studies (COREQ) was made use of (Additional file 2). Finally, the integrative phase/analytical stage involved a re-examination of qualitative findings in the field in light of existing literature and an attempt was made to establish a relationship between qualitative findings in the field, findings from the literature review and clinical practice [16].

### The theoretical phase

**Fig. 1:**
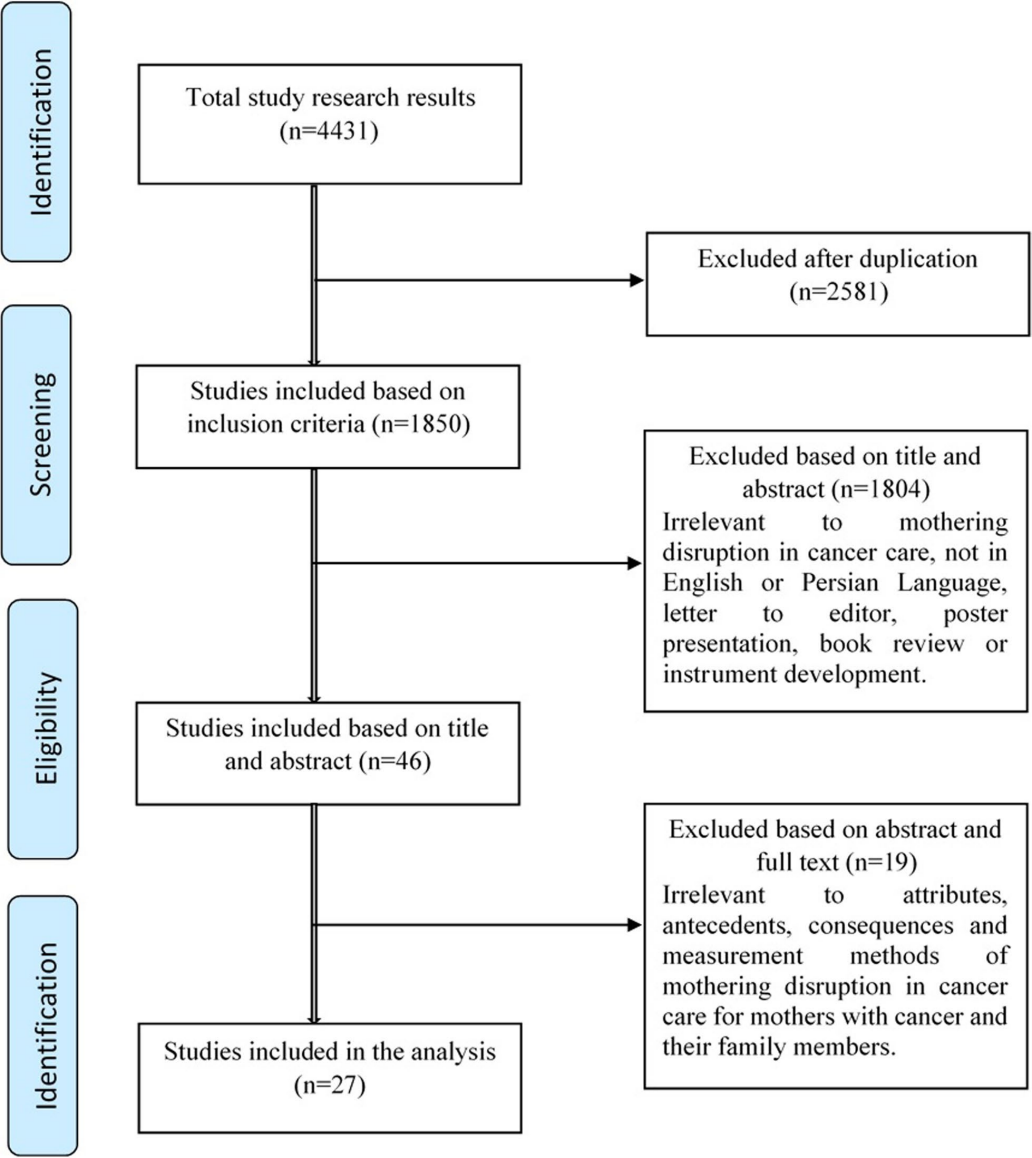
Flowchart of the study selection process of the concept analysis

At the beginning of this phase, a search of the literature was conducted in databases including PubMed, Science Direct, Scopus, ProQuest, Google Scholar, CINAHL, Wiley, Ovid, Magiran, and SID from 2000 to 2020 with the following keywords: *disrupted mothering/parenting*, *disrupting maternal /motherhood* and *breast cancer*. As articles in the cancer/breast cancer setting were scanned, the search was broadened by using the main terms, ‘*disrupted mothering*’ and ‘*care for children*’. Reference lists of identified studies were reviewed to include all relevant studies (hand searching). Keywords included: “*Mothering disruption*”, “*Disruption in mothering role*”, “*Disrupting Maternal*” OR “*Motherhood*”; Breast cancer: "*Breast Neoplasm*" OR "*Breast Tumors*" OR "*Malignant Neoplasm of Breas*t" OR "*advanced breast cancer*" OR "*breast cancer recurrence*". Article inclusion criteria was for relevant original articles and reviews articles which included the keywords in their title or abstract and papers with full text. We excluded non-English and non-Persian Language papers and papers discussing development of instrument (2 articles). Overall, 1850 abstracts were reviewed and eventually 27 studies (Table 1) were entered in the analysis (Fig. 1).

**Table 1:** Summary of the literature (N = 27)

Authors, year	Country	Study design	Sample	Key findings related to the attributes of disrupted mothering
Billhult and Segesten, 2003 [17]	Sweden	Phenomenology	10 women with no recurrent breast cancer	Balancing between being needed and perhaps not existing Balancing between own demands: Being strong and surviving, and being a good parent Balancing between telling the truth and protecting the children Strategies used by mothers with breast cancer: Gaining strength and support, Turn into positive, and continuing everyday life
Power, 2012 [9]	USA	Qualitative methodology	27 women Australia or America	Revealed how illness impacted upon the women‘s maternal lives, mothering activities and treatment decisions Encompasses their experiences with health professionals, as well as the way being hospitalized affected mother’s interaction with children The Supporting Cast ‘makes explicit women‘s main sources of support, namely their partners, female friends and relatives and others Reviewing the Performance’s details the quality of mothering
Vallido et al., 2010 [1]	Australia	Narrative synthesis	13 papers	Mechanism of disruption; Reframing the mother role, Protecting the children, Experiencing guilt or shame, Problems with healthcare professionals and living to mother, mothering to live
Semple and McCance, 2010 [18]	United kingdom	Systematic review	13 papers	Being a good parent Telling the children Maintaining routine at home
Wilson, 2007 [2]	Scotland	Narrative analysis	12 Women	Need of to survive and to protect their children Represented a fundamental re-formulation of their identities as mothers Biographical disruption while paradoxically also containing elements of biographical reinforcement
Tavares et al., 2018 [19]	Portugal	A mixed‐method systematic review	21 papers	Decision‐making processes about sharing the diagnosis with their children Mother‐child relationship and parenting after mother's diagnosis
Elmberger et al., 2005 [3]	Sweden	Grounded theory	10 mothers	Redefining oneself as a mother’, Interrupted mothering Facing the life-threatening illness and children’s reactions Striving to be a good mother; attempting to deal with moral responsibility as mother
Elmberger et al., 2008 [4]	Sweden	Qualitative secondary analysis	9 mothers with breast cancer	Becoming exhausted Facing it Finding meaning Becoming aware of the need for information and support Looking to the future Becoming energized
Elmberger et al., 2000 [20]	Sweden	Grounded theory	9 women with breast cancer	The main theme was transforming the exhausting-to-energizing process in being a good parent in the face of cancer
Campbell-Enns and Woodgate, 2013 [21]	Canada	Grounded theory	8 mothers with cancer	The meaning that mothers made of decisions maintain the mother child bond The conditions of the mothers’ lives influenced the meaning mothers assigned to decisions Coping strategies to facilitate maintaining the mother child bond in times of distress
Portis, 2008 [22]	U.S.A	Grounded theory	7 mothers	coping styles and communication, denial, loss of role/sense of self, communication with children, balance, breast cancer in context biographical disruption, the importance of community support, and living with uncertainty
Arida et al., 2019 [23]	U.S.A	Secondary analysis of focus groups	9 women	Evolving self-identities from healthy mother to cancer patient to woman mothering with cancer How motherhood was impacted by symptoms, demands of treatment, and the need to gain acceptance of living with cancer
Rashi et al., 2015 [24]	Canada	Qualitative, descriptive design	5 mothers and 7 fathers with a first cancer diagnosis	a Parental self-activated strategies; maintaining child routines, selective disclosure, strength and positivity, adapting to illness-related physical changes, and connecting with others who are similar b Tangible social networks; that meet transportation, child care, meal care, and psych emotional support needs c Suggestions to enhance person- and family-centered care; information to benefit the children, coordination of appointments, optimizing timing for support services, and the need for more tangible support
de Castro1 et al., 2018 [25]	Brazil	Exploratory qualitative design	10 mothers	Being afraid of death/cancer recurrence and leaving their child orphan Changing the values/meaning of life after illness Changing the family routine/child routine Having conflict/defeat/ambivalent feelings Having difficulties to attend their children
Mazzotti et al., 2012 [26]	Italian	Qualitative approach	8 women	Handle disease as a temporary event Detachment from their children, in an attempt to protect them Sort or long term changes in the life of the mother and family members The behaviors examined, adopted to safeguard children’s wellbeing create or enhance dysfunctional and paradoxical communication strategies
Kuswanto et al., 2018 [27]	Germany	Systematically review Meta-Analysis	30 papers	Psychological impact of cancer on mothers Changes in maternal identity and role Relationship changes and concerns for their children Meaning-making in cancer
Helseth and Ulfsæt, 2005 [28]	Norwegian	Exploratory design	18 parents	Living in a state of emergency Cancer was consuming their energy, physically and emotionally Striving to be good parents Shift of Priorities and change of values that often brought family members closer together Facing with challenges of illness by making the best of it Putting the needs of the children in focus and trying to maintain normal family life protecting the children and make the illness situation as secure and normal
Bekteshi and Kayser, 2013 [29]	U.S.A	Grounded theory	29 mothers	Closer relationships with their daughters, emotions such as fear, anger, or guilt Mothers were able to work through these emotions with their daughters through four relational competencies: a anticipatory empathy sensitivity about the impact of cancer on each other; b authenticity full presence without fear of abandonment; c mutual empathy caring and emotional support; d mutual empowerment capacity to empower one another
Baltisberger, 2015 [30]	U.S.A	Mixed methods	31 women	Keeping life the same while weathering cancer treatments; learning, adapting, accepting support, growing and normalcy
Öhlén and Holm, 2006 [31]	Sweden	Hermeneutic phenomenology	9 women	Facing Transformative Life Situations Being unreal Seeking and finding rhythm in the family Inability to maintain the role of a women Being confronted with irresolvable dilemmas Meaning in sever suffering Trying to maintain ordinary life Ambivalence: desire to maintain used to be ordinary and to face Changes in life Affirming a yearning for and seeking something new Remaining in the new and widening the perspective on everyday life
Bell and Ristovski-Slijepcevic, 2011 [32]	Canada	Ethnography	6 women	Mets and children: the hierarchy of suffering The need to maintain a sense of normalcy ”cram parenting” and “making memories”: Temporal disturbance in women’s of mothering
Walsh et al., 2005 [33]	U.S.A	Cross- sectional	204 women with breast cancer	Mother’s relationships with her children Emotional distress Increased closeness Role shift
Noorisanchooli et al., 2018 [34]	Iran	Qualitative method with conventional content analysis approach	12 patients with breast cancer	Mutual supports, mutual sufferings, and ambivalent feelings Mutual involvement of the patient and family with the disease
Kirsch et al., 2003 [35]	U.S.A	Inductive content analysis	4 couples	To protect their children from being frightened and worried
Rayson and Ruedy, 2001 [36]	Canada	Case study	A 32-Years-old woman	Resolving the conflict between two moral demands: being strong and surviving, and being a good parent
Shrestha et al., 2019 [37]	Nepal	Walker and Avant’s method	25 papers	The antecedences of maternal role; pregnancy, maternal identity, maternal behaviors, and adjustment to child-rearing, and bonding with the child The attributes; considered nurturing, protecting, caretaking, and managing household affairs The consequences; the awareness of neonatal status, balanced fulfillment of multiple roles, maternal role strain, and role conflict
Shin et al., 2008 [38]	Korea	Rodgers’ method of evolutionary concept analysis	54 papers	Attributes of maternal sensitivity; dynamic process involving maternal abilities, reciprocal give-and-take with the infant, contingency on the infant’s behavior, and quality of maternal behaviors

The studies were screened for eligibility (selection process), and title and abstract screening were undertaken by the first author to identify publications that did not meet the inclusion criteria. The first and second authors independently screened the full texts of the selected publications to match the eligibility criteria. The following data were extracted: author name, publication year, region, sample size, study methodology and key findings as antecedents, attributes and consequences of mothering disruption. For this purpose, the selected articles were read several times and independent of each other by all authors in order to extract appropriate codes, searching for words and phrases that related to disrupted mothering. Codes were reviewed, extracted into several categories such as antecedents, attributes and consequences of mothering disruption concept [12]. The results were then reviewed by three experts in fields of women health care and concept analysis to confirm authors’ perspectives on the coding and data clustering.

### Fieldwork phase

A qualitative conventional content analysis approach was used to explore the experience of Iranian mothers with BC. Following ERB approval, participants were recruited using purposive sampling. The twenty participants eligible for the study were those who met the following inclusion criteria: had a confirmed diagnosis of BC, attended the oncology center of Shahid Ghazi Tabatabaei Hospital, had children 16 years and/or younger who were living with the participant and had deep and rich experiences about mothering during BC and its treatment. Participants were mostly aged 35–49 years, had completed a course of initial treatment for BC in the last one year and were also at the follow-up stage without any signs of recurrence. Methods to ensure the protection of human rights, including the voluntary nature of participation were discussed with participants and oral and written informed consent was obtained from them at the beginning of the interview. Data were collected by the primary researcher. Participants were interviewed at a place of their choice, with each interview audiotaped and lasting 35–80 min. The interview continued until data saturation.

The interview guide developed for this study is provided as Additional file 3. The example of questions asked the participants included “Based on your own experience of motherhood, what comes to your mind, when you hear about the disrupted mothering in childcare?”, “What happens in the care of your children that makes you feel your role as a mother is impaired?”, and “If your role as a mother was to be impaired, what do you think would be the outcome, for you and other family members?”. The transcripts were analyzed with conventional content analysis following guidelines by Graneheim and Lundman [39] and analysis process included open coding, creating categories, and abstraction. Likewise, the recorded data were transcribed and attributes and important characteristics of disrupted mothering were extracted. The MAXQDA version 10 (software) was used for data management. In order to validate the trustworthiness of the data, the interviews’ transcripts and data interpretations were provided to three mothers with BC to compare the results with their own experiences (Member check). The peer debriefing method was also used. Thereafter, the analysis tables were discussed in weekly meetings with tutors and advisors (4 people) with sufficient skills in qualitative studies to ensure that the data analysis and interpretation is objective-orientated and are performed based on a logical and systematic approach. Codes and categories were sent to four researchers outside of research team to express their opinions concerning their credibility.

### Integration phase

Finally, this analytical stage involved a comparison of findings from the field with findings that emerged during the theoretical phase (the existing literature) by the authors who sought to establish the relevance of findings to clinical practice.

## Results

### Theoretical phase results

After reviewing the literature, the following definitions surfaced regarding the meaning of disrupted mothering. Disrupted mothering, has been defined as a woman perceiving that her maternal life has become disordered [1]. Letteney referred to disrupted parenting in women‘s caregiving capacity [40]. In the study by Grant et al., disruption referred to periods of not having custody of their children. However, disruption to mothering can take many forms and does not necessarily mean physical separation [41]. Wilson argues that with illness, a mother’s ability to care for her children is disrupted, a situation called biographical disruption [2]. Becker defined disruption as a part of the human condition. Disruption is therefore viewed as chaotic as it makes it difficult to fulfil cultural ideals and ideologies. Furthermore, the inability to fulfil these ideals threatens a person‘s ability to see themselves as a moral being [42].

In the Table 1, the studies reviewed are listed along with the author’s name, publication year, region, sample and its size, methodology of the study, and Key findings related to the attributes of disrupted mothering. The following attributes of disrupted mothering were gleaned from the literature search:

### Disturbance in maternal identity and roles

These include evolving self-identities from healthy mother to patient (23), and loss of role/sense of self and self-preservation (24). Mercer viewed maternal identity as the end point of the process of role attainment and articulated the stages of role attainment (anticipatory, formal, informal, and personal) during which maternal role behaviors evolve [43]. In the personal stage, the mother acquires confidence and competence in the performance of her role, thus establish the maternal identity [44]. However, because the continuous evolution of the mother’s personality in the face of life challenges in child-rearing which may erode mother’s self-confidence in her ability to rear her children (hence the ‘disruptive mothering’), Mercer proposed that the term, ‘Becoming a mother’ more aptly defines the constant change in the maternal identity [43].

The literature search revealed various concepts of the maternal role. Maternal role was defined as the behavioral responses to the expectations from a new mother and the mother’s perception of responsive mothering [45]. In another, maternal role was defined as a process that help the mother achieve competence and integrate the mothering behaviors into an established role in a way that makes her feel comfortable with her identity as a mother. The search also revealed that maternal role has two components, child care, and cognitive-affective activities, the latter which includes motherliness and attitudes of tenderness, awareness, and concern for the child's needs and desires [43, 46]. Also, the defining attributes of the maternal role were considered to include nurturing, protecting, caretaking, and managing household affairs [37].

#### Maternal insensitivity and unresponsiveness

Maternal responsiveness is defined as promptness or frequency of response to the infant’s signals [47]. However, responsiveness to infant’s cues can be considered as one aspect of maternal sensitivity and maternal responsiveness has been used interchangeably with maternal sensitivity [48]. Maternal sensitivity has been identified as a dynamic process involving a mother’s responses following perception and interpretation of her infant’s cues. The literature search revealed that there may be some subtle differences between maternal responsiveness and sensitivity. Maternal responsiveness has been described as responding to infants’ physical and emotional needs, while maternal sensitivity includes maternal behaviors that show sensitivity even to infants’ mental states [49]. It is stated that the most important factor distinguishing maternal responsiveness from maternal sensitivity is the absence of any qualitative aspect of the mother’s behavior and only promptness or frequency of the mother’s responses contribute to responsiveness; whether those behaviors are appropriate or not is not considered a focus of maternal responsiveness [47]. On the other hand, maternal sensitivity takes into account the quality or appropriateness of maternal responsiveness to the infant [50]. Thus, findings of this literature review would attest to the fact that disruption of the mothering role is akin to a lack of maternal responsiveness and maternal sensitivity as it can be equated to failure to respond to the behaviors of children and disruption in the process of child care”.

#### The career disruption process

Mothers with BC are distressed by the times, when their illness, through hospitalization and treatment, fatigue or disability, prevents them from caring for their children as they normally would. The prognosis, treatment and nature of the diseases means that the disruption is experienced in different ways, from having less energy for caring for and playing with children, to physical separation and increasing disability, future hospitalization or impending death. Mothering could be disrupted even when women were physically present with their children [20].

#### Biographical disruption

Critical event is a disruptive experience in the structure of everyday life, and this experience may be labelled as a form of biographical disruption. Biographical disruptions refer to the cessation of progress in the life of an individual by reason of critical illness [51]. The concept of biographical disruptions is replete in the literature. Cancer is known to alter an individual’s identity [52] and for a health crisis like BC, biographical disruption has been described as comprising three dimensions: the body, conceptions of self, and time. It is also a concept that affects early BC women largely in the post-treatment period with women fearing reoccurrence and altered physical forms [53].

### Fieldwork phase results

According to the analysis of the transcribed interview data, three main categories emerged for defining the mothering disruption concept. In the Table 2, the main categories are listed along with their attributes, codes and quotes. These main categories are explained below.

**Table 2:** Fieldwork phase results

Main categories	Attributes	Open codes	Quotes from participants
Reduced maternal sensitivity	1. Decline of belief in good mothering ability 2. Doubts about the adequacy of the physical and mental capacity to take care of the child 3. Dual emotions of being able or enable to take care of the child 4. Mother's physical and emotional unavailability 5.Not responding to children's needs	Dissatisfaction with the condition of the children (in the early stages of illness) Confusion about responding to the role expectations The separation of child from mother due to the impatience caused by complications of treatment Frequent referrals to follow up the disease Impatience and the inability to love children Lack of attention to details in care for child Indifference to children's educational affairs	"The disease made me bored to do the children's affair, before my disease I was doing them enthusiastically, but now it seems like a wave is coming and it will disappear my strong sprit." (Participant 7) "At the beginning of the illness, everything was meaningless for me. The joy of taking care of the kids and helping them to do their homework was not interesting for me." (Participant 5) "At the early stages of illness, I was just thinking about myself, I had even forgotten my children, and I was unaware of what they ate, wore, and did at home." (Participant 4) "I am injecting ampoules and I go to the clinic every three months to check on my condition. I am currently under the supervision of a doctor and immunotherapy. This morning I got up at 6 am, then I told my husband that I am tired of this illness and he tried to make me calm.” (Participant 2) “My daughter used to tell me all that happened at school after she came back, but now my impatience has made her unable to talk to me much and she distanced herself from me."(Pparticipant 17) "When I get lethargic and my physical problems overwhelm me, I can't tolerate anyone. After the illness, I didn't have the patience to love others as much as I used to, because I'm not in a good mood due to my illness." (Participant 14) "The importance I assume to some of the details is lessened because of my physical weakness, I'm not obsessed with doing homework and taking care of my children. In past I used to paint and play with my children but now I don’t."( Participant 10)
The imbalance between roles	1. Simultaneously taking care of yourself and your child 2. The difficulty of coordinating self and child-care activities 3. Simultaneous roles with illness	Simultaneous role of illness and being employed The responsibilities of a single parent living simultaneously with the disease Responsibility of providing the expenses of a single parent family simultaneously with the illness The multitude tasks of self and child-care with special circumstances The duties of a woman as a mother and wife simultaneously with illness Interference of disease follow up and care for children affairs	“I worked for patients of welfare state before my illness as a welfare worker, and I am still working for them, and this has made my responsibility much more." (Participant 20) "Shortly after I separated from my husband, I became ill and took on the responsibility of a single-parent family, including expenses of my illness and my family, on the other hand, the burden of illness increased my workload." (Participant 17) "My little son is mentally retarded and it takes a lot of time to look after him since I got sick and went to the doctor regularly, looking after my nutrition, medication and time management for doing my affair is very hard." (Participant 6) "As a mother of two four- and five-year-old children, I have a lot to do for my children, like cleaning up, feeding and taking them to the kindergarten and etc. On the other hand, I have to take care of my husband, and when I get home from work, I don't want to be frustrated, but since I got this problem (breast cancer), my job and taking care of my family have involved me." (Participant 2) "Every three month I have to go to the clinic to check my disease and between these intervals, if I have a problem, I will go to the doctor and this wastes my time and I can't make lunch for my children and husband." (Participant 6)
Role failure	1. Inefficiency in maternal role duties 2. Lack of quality in care for child 3. Failure in playing the role of education and socialization 4. Inability to protect the child 5. Inability to play a caring role 6. Inadequacy in mothering expectations as a good mothering	Decrease of ability to take care of children prior to illness Not completing the works Breaking down child care practices (children's school and recreation programs) Wasting time of childcare that caused by follow-up treatments Lack of interaction with children Not accompanying children in social situations Neglecting the nutrition and hygiene of children Inability to regulate children and home affairs	"I go out with the passion to do what I need to do, but after a while my body gets tired and I feel unable to finish my work even for cleaning the children's bedroom ….” (Participant 9) "The disease has ruined my previous way of life, the cancer has changed my life, I can't use my right hand to do things, because of surgery, I can't do the heavy work and I have to ask for help to do my work… I used to accompany my daughter to the door for going to school, but now I can't ….”(Participant 19) "Getting involved with the illness and hospitalization for surgery and chemotherapy left me unaware of the kids and now I have to waste a lot of time for my disease follow up, because I go to another city for my treatments, so I don't have enough time to be with my children." (Participant 15) "I can't talk to the kids calmly and without fear, and I am unable to provide the time they need to have a normal life. I haven't been able to talk to my kids much since I got sick. I do not have the patience to love the children because I'm not in mood as always due to my illness."(Participant 3) "Sometimes I have a severe headache that I can't go out with my kids for fun or party too much and they complain about it. After my illness I can't spend enough time with my children." (Participant 8) “The drugs I use have made me less energetic, and I can't cook good food for children. Sometimes my kids' affairs and my housework get messed up as if I have no more control over things.”( Participant 13)

### Reduced maternal sensitivity

Mothers with BC perceived their health status differently than healthy mothers and expressed their doubts about the adequacy of care for their children due to their physical and mental (anxiety and depression) challenges, thus giving rise to a decline in the belief in good mothering ability. The authors perceive this to be a form of reduced maternal sensitivity. Reduced maternal sensitivity or maternal insensitivity is the inability of mothers to respond to behaviors arising from children's needs. Mothers reported that they sometimes felt incapable of caring their children as any ideal mother would, and the conflicting feelings of being able or unable to care for children made them physically and mentally exhausted the more. They also reported that in a bid to pursue rigorous and aggressive treatment regimens and to overcome fatigue of BC by having long rests, they became physically and emotionally detached from their relationship with their children and thus were unavailable to respond to their children's needs.

### The imbalance between roles

Another concept that emerged from the interviews was an imbalance between roles as typified by the mothers’ accounts of juggling the role of self-care with child care. The mothers reported that in the course of the illness, they had disease-related needs that had to be met in order for them to be able to continue their maternal role and for their health to be restored to a reasonable quality. Many reported being faced with issues like fatigue, disability, and motor limitations, added to the need to complete their treatment regimen. This often clashed with the expectations of the society in which they found themselves. Some mothers in this study admitted to trying to prioritize their children’s needs with regards to maternal duties, but physical disabilities and symptoms of the disease often made it difficult for them to coordinate self-care and child-care activities. Others complained of being emotionally impaired from being employed and having multiple roles at the same time, such as attending to spousal need, motherhood and illness, thus making the role of mother tedious and less attractive.

### Role failure

In discussing maternal role failure, inefficiency in executing maternal roles was cited by many as a cause of role failure by some mothers. Many of the mothers reported being burdened by numerous duties which included education and protection of children in a background of illness and unpleasant treatment complications, the latter often making it difficult for such mothers to have regular access to their children. Thus, a reduction in the mothers’ interactions with their children led to poor quality of care for child. Consequently, mothers were seen by family members and neighbors alike as having failed to meet expectations of maternal role, in terms of education, socialization, and protection as a good model of motherhood (responsibility, responsiveness and attention to physical and psychological needs of children).

### Integration phase results: integrated definition of mothering disruption concept

After analyzing all aspects of the concept using a hybrid model and based on all the two phases discussed above, The authors classified the findings of the previous two stages into three categories to explain the integrated definition of mothering disruption, which included (1) disease as threating maternal role and identity, (2) inability to interpret and respond to child behavior, and (3) support for transitioning from being patient toward maternal competency.

Authors arrived at the following conclusions about the opinions of this cohort of Iranian mothers with BC regarding disrupted mothering. In their opinion, the overbearing and difficult situation they faced arising from the illness and its treatment combined with the continuation of the mothering role threatened their maternal role and identity. Also grossly affected was their ability to interpret and respond to their children’s behaviors and needs, especially the dependent ones, even when they tried to ignore the burden of the illness and ‘being a patient’ and just carry out their mothering role. Thus, mothers need the help of people around them for support as they transition from being sick to recovering and being able to playing theirs roles as mothers (Table 3).

**Table 3:** Theoretical, fieldwork, and integration phases results

Theoretical phase	Fieldwork phase	Integration phase	
		Category	Subcategory
Disturbance in maternal identity and roles	Reduced maternal sensitivity	Disease as threating maternal role and identity	1. Unbalancing in roles as identity threat of mothering 2. Waiver of duties other roles to achieve balance in mothering role 3. Disease as imposing an unwanted role
Maternal insensitivity and unresponsiveness	The imbalance between roles	Inability to interpret and respond to child behavior	1. Apparently little communication but actually fighting for each other 2. Physical and emotional disrupted 3. Disease as consuming mother’s energy 4. Tired of taking care yourself and children 5. Mothering under negative emotions from fear to shame
The career disruption process	Role Failure	Support for transitioning from being patient toward maternal competency	1. Dependence on role playing 2. Disruption as an opportunity to restore role 3. Decreased capacity role playing 4. Need to support to mother’s role playing 5. The Supporting cast: maintenance of maternal responsibility by supporting their children
Biographical disruption			

## Discussion

Mothers with BC struggle with physical, mental, and social challenges after diagnosis and cancer treatment and this negatively impacts on their family life as many are unable to fulfil their mothering role subsequently [54] as they are caught in-between two conflicting roles or identities: the pre-diagnosis identity of mother and the post-diagnosis identity of ‘patient’ [55]. While some research has been conducted on the experiences of mothers with various illnesses in general and cancer in particular, and how these impact on their roles as wives, mothers and homemakers [1, 32, 56], very little is known about this from the perspective of women living in Iran with BC. The traditional Iranian society is patriarchal [57], characterized by a strict division of labor between men and their wives where the daily administration of household issues, care of children and training of female offspring for their future domestic roles is the exclusive preserve of the mother and the father plays an authoritative-directive control role [58]. Thus, this findings from study are pertinent as they revealed the relationship between mothering and cancer disruption, a situation termed ‘disrupted mothering’, among Iranian women and will provide culturally-sensitive approach by clinicians to the care of these women. In this study, three constructs were identified: cancer disease threating maternal role and identity, inability to interpret and respond to child behaviors and needs, and support for transitioning from being a patient to maternal competency.

One aspect of the life of the woman with BC, which is her self-identity, is often under assault as a result of the disease and its treatment. Strickland has suggested following the cancer diagnosis, the mother finds herself unintentionally in a very difficult situation where she tries hard to be both a good patient and a dedicated mother [59]. The threat to maternal role and identity expressed by mothers in this study echoes the findings of Fisher and Connor where mothers felt confused, isolated, more like ‘objects’ and no longer like mothers once cancer treatment was commenced [54]. The loss of the mothering identity is further aggravated by having dependent children [60, 61]. Besides maternal competence, mental health of the mothers is often affected [28]. Majority of participants stated that the being a cancer patient was an unwanted role which led to the need for simultaneous self and child-care, and that the difficulty of coordinating and balancing these needs led to an imbalance between the roles. Also, mothers reported being forced to give up some of their duties in a bid to balance their maternal role, sometimes to the detriment of the children, a concern that is equally mirrored among Korean women with BC [62]. The opinions expressed by the Iranian mothers in this study also validates the assertions put forward by Bertero and Wilmoth following a meta-synthesis of qualitative research, that breast cancer affects the identity of women with BC irrespective of race or ethnicity [63]. Therefore, clinicians, especially nurses, should be aware of the conflicts that may arise in the identity of the woman with cancer so as to fashion suitable programs that will help such patients and support them as they transition between the role of mother and that of patient.

The second construct in this concept analysis was “the inability of mothers to interpret and respond to their children's behaviors and needs. The cancer diagnosis is known to affect patients’ psychology as well as their confidence in carrying out their parenting role [19]. It has been observed that at all phases of the cancer journey, mothers are constantly worrying about their children’s well-being and some even go to great lengths to keep the knowledge of the disease from their children [59, 64]. The narratives obtained when analyzing the issues that gave rise to this second construct attest to that observation as participants reported that being involved in illness and treatment had reduced the physical and mental energy they had previously to engage in simultaneous care of themselves and of their children. They also reported that reduced communication with children due to fatigue led to physical and emotional disruption in such a way that they were unable to understand their children's behaviors because they spent insufficient time with them and thus were unable to respond appropriately to their behaviors and needs. A cohort of Canadian mothers with cancer whose opinions were sought on this issue, divulged strategies to overcome their inability to respond to their children’s needs. Some of these strategies included making frequent phone calls, using Skype™ to talk with the children during periods of hospitalization, paying more attention to the children’s verbal and non-verbal communication cues to watch out for behavioral changes [64].

Many of the women felt they were not recognized as mothers, but instead were treated as ‘just a patient’ and had a lot of troubles fulfilling their responsibilities regarding the well-being of children and the stability of family life. This is the bane of the third construct of analysis which was “support for transitioning from being patient toward maternal competency.” This narrative affirms previous findings that point to the need for a strong support structure for women with BC during the entire disease and treatment continuum and beyond and nurses can address their concerns. Previous evidence suggests that the usual services provided in health centers do not fit the needs of mothers, as the diagnosis of cancer can be very destructive and complex, given the unique position of women in playing their role as mothers [59]. This is even more pertinent for women in the Iranian society who are burdened with the responsibility to organize the affairs of their homes. In this regard, they need the support of nurses in clinical and social settings [6]. Nurses can get acquainted with the attributions of the concept of mothering disruption, and help mothers in their transition from a sick mother to a healthy mother and performing their mothering duties. One way the authors posit this can be achieved is by exploring the women’s coping strategies by nurses. This may help the women make the transition between mother and patient back and forth, seamlessly. The previous studies have identified several largely positive coping strategies used by Iranian Muslim women with breast cancer which include acceptance of the disease as the will of God, intentional forgetfulness, active and passive acceptance, interaction with other patients with BC and spousal support or support from significant others. Such patients need to be encouraged by their healthcare providers to move to the point in their lives where they can live near-normal lives despite their predicament [65, 66].

Although this study possesses many strengths, it is not without limitation. This study was limited to patients who received follow-up at a single hospital. Therefore, their experiences might not be generalizable to the general population of Iranian mothers with BC. However, the authors consider that it will be relevant locally in Tabriz and similar regions of Iran.

## Conclusions

In sum, this study is significant in that it is the first time a study has proposed a conceptual definition of the mothering disruption of these mothers in Iran. It reveals that Iranian women with BC express the same fears and concerns regarding the disruption of their mothering roles and identities, as other mothers with BC globally. The definitions, attributes, antecedents, consequences, and integrated definition of mothering disruption identified in the present study can be used to evaluate mothering disruption in healthcare settings and develop theory-based interventions for decreasing this disruption in mothers with BC. This concept analysis provides information, which can be used in nursing practice, education, research, and management.

By analyzing the concept of disrupted mothering as conducted in this study, through the integration of narratives provided by the women with BC with the evidence from scientific literature, an opportunity has been created for healthcare providers in caring for Iranian women with BC to have a better understanding of the experiences of their patients in order to tailor the care and support these women receive to enable them retain their mothering identities, fulfil their mothering roles and still judiciously comply with all treatment decisions raised during their care in hospital and in the post-hospitalization period. While this study has attempted to fill the gaps in literature concerning disrupted mothering in the context of women in an Islamic culture, it also facilitates clinical and transferable knowledge in nursing performance and it can help nurses and other clinicians choose the appropriate interventions to help these mothers find a balance between self-care and other mothering roles.

Future research directions would include specific culturally-sensitive psychosocial interventions targeted at eliminating the disruptions in mothering and the development of tools to measure the level of disruption in mothering, by the healthcare providers to aid in early identification and prompt resolution of disruptions in the mothering role and identity. In addition, as the definition of disrupted mothering is likely to vary across treatment stages, further comparative research is needed to analyze the concept of mothering disruption in the early and late stages of breast cancer disease and treatment continuum.

## Supplementary Information

**Additional file 1**. The PRISMA Checklist for study.

**Additional file 2**. The COREQ Checklist for study.

**Additional file 3**. The interview guide was developed for the study.

## Data Availability

The datasets used and/or analyzed during the current study are available from the corresponding author on reasonable request.

## Abbreviation

BC: Breast cancer
