# Supplementary material for: Disrupted mothering in Iranian mothers with breast cancer: a hybrid concept analysis
Source: BMC Womens Health. 2021 Jun 5;21:234. doi: 10.1186/s12905-021-01346-w (PMC8178819; doi:10.1186/s12905-021-01346-w)
Supplement: Supplementary file 3 — Additional file 3. The interview guide was developed for the study. [file 12905_2021_1346_MOESM3_ESM.docx]

Supplementary file 3: The interview guide was developed for the study

| Participant IDNO \|__\|__\|__\|__\| Researcher Initials \|__\|__\|__\| Participant age\|__\|__\|__\|  Children number \|__\|__\| Participant job\|__\|__\|__\| Participant education\|__\|__\|__\|  Date \|__\|__/__\|__/__\|__\|  **Introduction**  I am ______________________________ from ______________________   1. General purpose of the study 2. Aims of the interview and expected duration 3. Who is involved in the process (other participants) 4. Why the participant’s cooperation is important 5. What will happen with the collected information and how the participant/target group will benefit 6. Permission for recording interview 7. Any questions? 8. Consent   **Warm up [demographic & health history]**  Can I ask some details about you and your disease?  Treatments received for breast cancer____________  Years elapsed of the breast cancer diagnosis \|__\|__\|yrs\|__\|__\|mths  Do you have any children under 16yrs old living with you now? □ Yes □ No  Number children under 16yrs old living with you now? ____________  Do you live with your spouse? ____________  Are you the head of the family? ____________  Your spouse's personal social information? ____________  **Now I am going to ask you some questions about your experiences as a breast cancer women.** | |
| --- | --- |
| **Domain** | **Topic and Probes** |
| Meaning experience of disruption in mothering role for participant? | Based on your own experience of motherhood, what comes to your mind, when you hear about the disrupted mothering in childcare?  How did the illness change the normal routine of your life and that of your children?  Probes: Tell me more about those changes. |
| Change in mothering role playing in the breast cancer trajectory | What happens in the care of your children that makes you feel your role as a mother is impaired?  Probes: What problems could you foresee and what ways do you think these might be solved? |
| Consequences of disruption in the mothering role for participants and their family member | If your role as a mother was to be impaired, what do you think would be the outcome, for you and other family members? |
| Support resources | Who and how helped you to fulfil your role as a mother in addition to treating your illness?  Probes: Can you suggest any ways that health care providers can support for you and your family to overcome to your problems in playing mothering role duties? |
| Social support | In your opinion, what do patients (mothers and children) expect from policymakers When they need more support due to the pressures of the disease and its treatment? |
| **Closing**  Is there anything else you think is important that we have not talked about?   1. Summarise 2. Thank participant 3. Provide extra information and contacts to participants | |
